# Health extension workers' level of job satisfaction in western Hararghe Zone, eastern Ethiopia: an institutional-based cross-sectional study

**DOI:** 10.3389/frhs.2024.1353072

**Published:** 2024-03-12

**Authors:** Kemal Aman, Tesfaye Gobena, Behailu Hawulte, Melat B. Maruta, Adera Debella, Addis Eyeberu, Rudwan Abrahim, Olifan Wakjira, Ibsa Mussa

**Affiliations:** ^1^ Tullo Woreda Health Office, Tullo, West Hararghe, Oromia, East Ethiopia; ^2^ School of Public Health, College of Health and Medical Sciences, Haramaya University, Harar, Ethiopia; ^3^ School of Nursing and Midwifery, College of Health and Medical Sciences, Haramaya University, Harar, Ethiopia; ^4^ Obstetrics and Gynecology, Menelik Hospital, Addis Abeba, Ethiopia; ^5^ School of Medicine, College of Health and Medical Sciences, Haramaya University, Harar, Ethiopia

**Keywords:** health extension workers, job satisfaction, Tullo, Hararghe, eastern Ethiopia

## Abstract

**Background:**

There is a concern that job dissatisfaction among health extension workers (HEWs) reduces the benefit of investment in the execution of health extension programs. Hence, the purpose of this study was to explore the level of job satisfaction and factors affecting it among the HEWs in the West Hararghe Zone, Oromia Regional State, eastern Ethiopia.

**Method:**

An institutional-based cross-sectional study was conducted among 416 randomly selected health extension workers from 20 September 2020 to 20 October 2020. A pretested, structured questionnaire was used to collect the data. STATA 14.2 was used for data analysis. Bivariable and multivariable binary logistic regression analyses were also performed. Statistical significance was set at *P* < 0.05.

**Results:**

The overall level of satisfaction of health extension workers was 51.8% [95% confidence interval (CI): 46.97%, 56.6%]. Earning more than 5,260 ETB as salary [adjusted odds ratio (AOR) = 1.69, 95% CI: 1.01, 2.85], working more than 10 km from the district town (AOR = 1.59, 95% CI: 1.01, 2.53), receiving supportive supervision (AOR = 1.64, 95% CI: 1.06, 2.55), and not living with parents (AOR = 1.94, 95% CI: 1.24, 3.04) were significantly associated factors with HEW job satisfaction.

**Conclusion:**

Nearly half of the health extension workers were dissatisfied with their jobs. Supportive supervision, compensation, distance, and parental home location were all predictors of job satisfaction. It is critical to establish intervention tactics that may satisfy and motivate HEWs to expand health coverage, strengthen health extension programs, and improve service delivery.

## Introduction

Community health programs enable vulnerable populations to access healthcare (1). To achieve equitable universal health coverage, there is an increasing need to understand how to best administer community-based health initiatives such as community health worker (CHW) programs (2). Health extension workers (HEWs) in Ethiopia are important components of health service provision at the community level.

The health extension program has had a real impact on rural people's beliefs and actions regarding illness prevention, family health, cleanliness, and environmental sanitation (3). The program's benefits include increased service utilization, improved awareness and care-seeking, increased latrine building and utilization, improved disease outbreak reporting, and a high degree of community satisfaction (4). Despite these successes, the health extension program faced challenges such as resource gaps, limited supportive supervision, a lack of a well-established referral system, high HEW turnover, a lack of a clear career structure for HEWs, an unattractive salary scale, inadequate medical equipment supplies of drugs for minimum curative services, budget, furniture, and vehicles, which were linked to a low level of job satisfaction and demotivation (5).

The term job satisfaction refers to the attitude and feelings of people about their work (6). Favorable attitudes toward the job indicate job satisfaction, whereas negative and unfavorable attitudes toward the job indicate job dissatisfaction (7). A high level of job satisfaction has a positive effect on workers' health-related quality of life (6, 8, 9), job performance (10, 11), retention in work (12), quality of healthcare delivery (13), and patient satisfaction (14, 15). Low job satisfaction may result in staff turnover, tiredness, absenteeism, undesirable job performance, and poor quality of service to clients (16).

Previous studies have shown that job satisfaction could be positively influenced by several factors, such as payment and compensation, good interpersonal relationships, training and career growth, supportive leadership, recognition by management, better teamwork, and a safe working environment (17–21). Conversely, job satisfaction could be negatively affected by factors such as work load, work‒family conflict, poor doctor‒patient relationships, improper supervision, a lack of training opportunities, low salaries, and financial rewards (22–24).

The degree of job satisfaction and its determinants among health professionals have been the subject of numerous types of research throughout the world and in Ethiopia. However, most of them concentrate on the satisfaction of professionals, such as pharmacy professionals (25), nurses, physicians, and laboratory professionals (26). Due to this, little is known about rural HEWs' job satisfaction despite their difficult working environment, the conditions under which HEWs operate, and their level of job satisfaction (27). To the best of our knowledge, there were few published studies related to job satisfaction among rural HEWs, except in the Hadiya zone reporting 52.7% (28), Sidama, 36.6% (29), and the eastern Showa zone reporting 16.6% (30). Anecdotal evidence also indicates that resource constraints and an underdeveloped environment influence satisfaction (28–30). So far, no study has been conducted in the West Hararghe Zone, eastern Ethiopia, particularly in the study area. Therefore, this study tried to explore the level of job satisfaction and factors affecting it among the HEWs in the West Hararghae Zone, Oromia Regional State, eastern Ethiopia.

## Methods and materials

### Study design, population, and setting

An institutional-based cross-sectional study was conducted among health extension workers working in health posts in the West Hararge Zone of eastern Ethiopia from 20 September to 20 October 2020. West Hararghe is one of the Oromia National Regional State's 21 zones, bordered by the East Hararghe Zone in the east, Somalia Regional State in the north, Bale Zone in the south, and Afar Regional State in the west. The zone was divided into 15 rural districts and 2 administrative towns. It is divided into 38 urban kebeles and 455 rural kebeles. In terms of healthcare, the zone has 5 hospitals, 85 health centers, and 454 rural health posts. A total of 1,028 HEWs labor in the zone's various districts and kebeles (31).

### Eligibility criteria

All health extension workers currently working in the west Hararghe zone were included in the study, while those who were on annual or sick leave, left for training, or refused to respond were excluded.

### Sample size determination and sampling procedure

The required sample size was determined using a single population proportion formula by considering a level of job satisfaction of 56.2% (32), a 5% marginal error, and a 95% confidence interval (CI). The calculated sample size was 378, and by adding a 10% non-response rate, the final sample size was 416.

A two-stage sampling technique was used to select the appropriate study population. First, 2 towns (Chiro and Badesa) and 7 districts (Rural Chiro, Meiso, Doba, Tulo, Boke, Gemechis, and Oda Bultum) were randomly selected from a total of 15 districts in the zone. Then, the study sample of health extension workers was selected randomly out of the total HEWs working in the selected districts (529). The calculated sample size was proportionally allocated according to the list of developed sample frame health extension workers.

### Data collection methods and procedures

The questionnaire was developed after reviewing related works of different literature, and some of the questionnaires were adopted from previous studies and contextualized accordingly. Data were collected using a pretested and structured interviewer-administered questionnaire, the tool includes sociodemographic data; organizational, behavioral, and motivational-related variables; and job satisfaction. The wording and sequence of questions were designed in such a way that the sequence of ideas (from general to specific and from easy to difficult questions) was maintained. To assure the data quality, four trained skilled health workers and one supervisor were used to collect the data, and the task was closely supervised by the investigators. First, we translated it while keeping the purpose of the questionnaire and the intent of the questions in mind. It was translated by group members who were bilingual experts. To ensure the translation's accuracy, the questionnaire was translated back into English by someone who had not seen the original version and was unfamiliar with the questionnaire's context. The back-translated version was then compared with the original, and any meaning differences were corrected. After that, to ensure cross-validity, we tried to interview a set of respondents in English and another set in the local language, such as Amharic; their answers were then compared to detect differences in understanding. Finally, pretesting was conducted to identify questions that were poorly understood, ambiguous, or elicited hostile or other undesirable responses. We attempted to conduct a pretest using the already-translated questionnaire. We tried to implement all the steps in pretesting, such as obtaining an evaluation of a questionnaire and testing the revised questionnaire through its paces with friends, colleagues, and so on. Statistically, we performed Cronbach's alpha, which is a measure used to assess the quality of our employed instruments. Regarding the Cronbach coefficients of each item, the scores were 0.92, 0.87, 0.90, 0.85, 0.88, and 0.91. The overall average score was 0.89, which was within acceptable ranges.

### Variables and their measurement

The dependent variable of this study was level of job satisfaction. Assessment of job satisfaction was measured by using 20 items each scored on a 5-point Likert scale with 1 denoting strongly dissatisfied and 5 denoting strongly satisfied with the Minnesota Satisfaction Questionnaire (MSQ) short form (33). The items such as salary and benefits, grievances and redressals, promotion, working environment, training, and administrative issues satisfied positive statements and vice versa for negative statements were prepared by reviewing previous similar studies (21, 34, 35). The overall job satisfaction was estimated by taking the sum score of all the subscales. Then, to measure the level of job satisfaction of each individual, respondents who scored more than half of the sum of all the satisfaction scale items were considered as satisfied with their job and those who scored half and below were taken as dissatisfied (21, 36). For each domain factor, the sum score of each variable under the value of 50 was taken as a cut point value to determine whether a health worker was satisfied with his/her job or not. As a result, healthcare professionals who scored a value of 50 and below were considered as dissatisfied and those who scored greater than 50 were considered as satisfied (36). The independent variables were: sociodemographic characteristics (age, religion, marital status, family residential place, and education level); organizational, behavioral, and motivational level factors (number of health post, work experiences, distance from the woreda town, recognition, ever received carrier development, supervision in the last 3 months, salary, frequency of supervision, and incentive); and alternative job profession category (6).

*Kebele*: Health extension package (HEP) service delivery is structured at the lowest level of governance in the nation, known as the kebele, which typically has a population of 5,000 people.

Based on the primary healthcare (PHC) tenet, the HEP is a collection of fundamental and mandatory promotive, preventative, and curative health services that are directed at homes in a community. It has 4 main program areas and 16 key health items.

*A health post* is a two-room building that houses most of the peripheral healthcare units. This is the initial level of healthcare delivery for the community, with a focus on preventive and promotional care.

### Data processing and analysis

The data were encoded, loaded into Epi-Data version 3.1, and exported to STATA version 14.2 for processing and analysis. Descriptive statistics, percentages, and summary tables were used to describe the results and the predictor variables. Multivariate logistic regression was used to assess the relationship between the dependent variable and independent factors. A 95% CI was also used to identify the important characteristics linked to the level of job satisfaction. A *P*-value of 0.05 was used to demonstrate statistical significance. The strength and direction of the connection between work satisfaction and related characteristics were assessed using odds ratios (ORs) and 95% CI. The multicollinearity of the explanatory variables was examined before running the model. Continuous variables were checked using the variance inflation factor (VIF), whereas the contingency coefficient was employed to check categorical independent variables.

### Ethical consideration

Ethical clearance was obtained from the Haramaya University, College of Health and Medical Science, Institutional Health Research Ethics Review Committee (IHRERC) by a formal letter with the reference number IHRERC/209/2020 on September 23/2020. Support letters from the university were written to the West Hararghe Zone Health Office. After getting all the permission letters from the responsible body, informed, voluntary, written consent was signed by the study participants. Confidentiality was maintained by using codes instead of the participants’ names. The participants were also informed that they had the full right to refuse to participate or withdraw at any time from the research. All methods were performed according to relevant guidelines and regulations in the Declaration of Helsinki.

## Results

### Sociodemographic characteristics

A total of 415 HEWs participated in this study, with a response rate of 99.7%. The mean age of the respondents was 28.7 years (SD ± 0.19). Of the total health extension workers, 370 (89.2%) and 244 (58.8%) were married and attended level III education. Two-thirds of the HEWs were working out of their family residences (Table 1).

**Table 1:** Sociodemographic characteristics of health extension workers at health posts of West Hararghe Zone, eastern Ethiopia.

Variables	Number	%
Age in years		
18–25	88	21.2
26–30	224	54.0
>30	103	24.8
Religion		
Muslim	208	50.1
Orthodox	180	43.4
Protestant	27	6.5
Marital status		
Married	370	89.2
Unmarried	45	10.8
Living with parents		
In the kebele	141	34.0
Outside of this kebele	274	66.0
Educational level		
Level III	244	58.8
Level IV and above	171	41.2

### Organizational, behavioral, motivational level factors

We found that the majority of respondents, 305 (73.5%), had served at more than one health post. The mean service years of the responding HEWs were 2.65 years (SD + 0.69), with 318 (76.6%) having more than 5 years of service. About 275 (66.3%) of the HEWs were serving in health posts at a distance greater than 10 km from their district's main town. Most respondents [264 (63.6%)] were not recognized by their organizations. More than half [220 (53.0%)] of the HEWs did not undergo carrier development. Almost 217 (52.3%) HEWs received supervision in the last 3 months. Furthermore, the current salary in ETB was 208 (50.1%) greater than or equal to the 5,260 birr, and 358 (86.3%) received supervision on a quarterly basis and above. The majority [377 (90.8%)] responded that they did not receive incentives except for the regular monthly salary paid to them (Table 2).

**Table 2:** Organizational-related factors of health extension workers in west Hararghe Zone, eastern Ethiopia.

Variables	Number	%
Number of health post-HEW served		
1 HP	110	26.5
≥2 HPs	305	73.5
HEW service year		
≤3 years	50	12.1
4–5 years	47	11.3
>5 years	318	76.6
Distance from woreda town		
≤10 km	140	33.7
>10 km	275	66.3
Recognition*		
Yes	151	36.4
No	264	63.6
Ever received carrier development		
Yes	195	47.0
No	220	53.0
Received supervision in the last 3 months		
Yes	217	52.3
No	198	47.7
Current salary in ETB		
≤5,260	208	50.1
>5,260	207	49.9
Frequency of supervision		
Two months and below	57	13.7
Quarterly and above	358	86.3
Incentive*		
Received	38	9.2
Not received	377	90.8

Recognition (Certificate, Letter of recognition, and other awards), incentives (peridium, Certificate Letter), ETB, Ethiopia birr; HP, health posts.

### Level of job satisfaction

**Figure 1:**
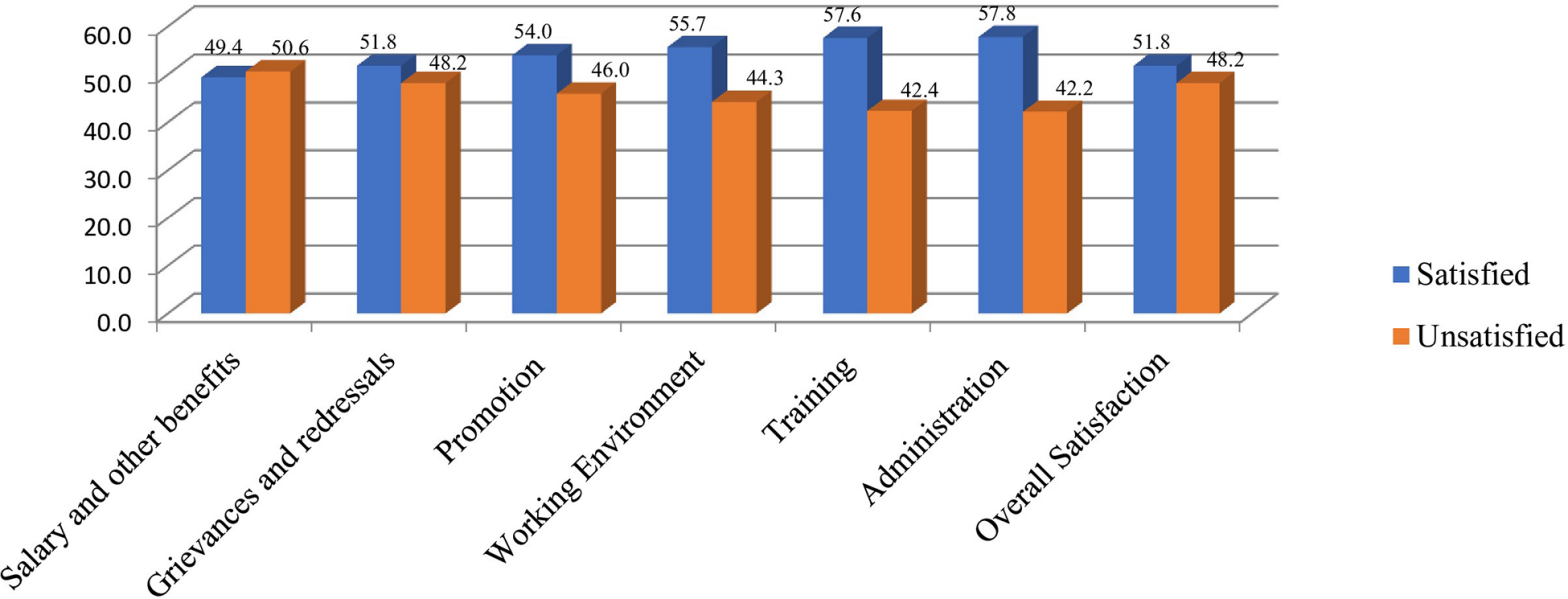
Level of job satisfaction of health extension workers in West Hararghe zone, eastern Ethiopia.

Of the total participants, 49.4%, 51.8%, 54.0%, 55.7%, 57.6%, and 57.8% were satisfied with their salary, grievance and redressals, promotion, working environment, training, and administration, respectively. The overall satisfaction level of HEWs was 51.8% (95% CI: 46.97%, 56.60%) (Figure 1).

### Factors associated with job satisfaction

In the bivariable analysis, predictor variables such as age, religion, marital status, recognition, education, distance, salary, number of health post (HP), service year, supervision, living with parents, frequency of supervision, and incentives were significantly associated with job satisfaction. However, in the final model of multivariable logistic regression analysis, predictor variables such as age, marital status, education, distance, salary, number HP, service year, supervision, and living with parents were significantly associated with job satisfaction.

A binary logistic regression model was used to identify factors associated with the outcome variables. Accordingly, from the variables in the multivariate analysis, earning more than 5,260 ETB as salary, living more than 10 km from the district town, receiving supportive supervision, and living with parents were significantly associated with job satisfaction.

Health extension workers working who got a monthly salary >5,260 ETB were 1.69 times more likely to be satisfied [adjusted odds ratio (AOR) = 1.69, 95% CI = 1.01, 2.85] than those who received greater than 5,260 per month. Health extension workers working at a distance of more than 10 km from their district town were 1.59 times more likely to be satisfied (AOR = 1.59, 95% CI = 1.01, 2.53) than those who worked in health posts less than 10 km from the district town. Health extension workers who received supportive supervision were 1.64 times more likely to be satisfied (AOR = 1.64, 95% CI = 1.06, 2.55) than those who did not receive supportive supervision from near the health center. Health extension workers not living with parents were 1.94 times more likely to be satisfied (AOR = 1.94, 95% CI: 1.24, 3.04) with their jobs compared with those living with their parents (Table 3).

**Table 3:** Factors affecting level of job satisfaction among health extension workers in West Hararghe Zone, eastern Ethiopia.

Variable	Satisfied, *n* (%)	Unsatisfied, *n* (%)	Crude OR (95% CI)	Adjusted OR (95% CI)
Age				
18–25	49 (22.8)	39 (19.5)	1	1
26–30	117 (54.4)	107 (53.5)	0.87 (0.53, 1.43)	0.80 (0.39, 1.63)
>30	49 (22.8)	54 (27)	0.72 (0.41, 1.28)	0.71 (0.32, 1.62)
Educational level				
Level 3	118 (54.9)	126 (63)	1	1
Level 4 and above	97 (45.1)	74 (37)	1.39 (0.94, 2.07)	1.58 (0.96, 2.60)
Distance				
≤10 km	61 (28.4)	79 (39.5)	1	1
>10 km	154 (71.6)	121 (60.5)	1.65 (1.09, 2.48)	1.59 (1.01, 2.53)*
Salary				
≤5,260	98 (45.6)	110 (55.0)	1	1
>5,260	117 (54.4)	90 (45.0)	1.46 (0.99, 2.15)	1.69 (1.01, 2.85)*
Number HP				
1	65 (30.2)	45 (22.5)	1.49 (0.96, 2.32)	1.74 (0.99, 3.05)
≥2	150 (69.8)	155 (77.5)	1	1
Service year				
≤3	29 (13.5)	21 (10.5)	1	
4–5	25 (11.6)	22 (11)	0.82 (0.37, 1.84)	0.82 (0.35, 1.99)
>5	161 (74.9)	157 (78.5)	0.74 (0.41, 1.35)	0.79 (0.30, 2.08)
Supervision				
Yes	126 (58.6)	91 (45.5)	1.69 (1.14, 2.50)	1.64 (1.06, 2.55)*
No	89 (41.4)	109 (54.5)	1	1
Living with parents				
Yes	124 (57.7)	147 (73.5)	1	
No	91 (42.3)	53 (26.5)	2.04 (1.34, 3.08)	1.94 (1.24, 3.04)*
Marital status				
Married	188 (87.4)	182 (91)	1	1
Unmarried	27 (12.6)	18 (9)	1.45 (0.77, 2.72)	0.89 (0.453, 1.82)
Career structure				
Yes	107 (49.8)	88 (44)	1.26 (0.85, 1.85)	1.06 (0.66, 1.69)
No	108 (50.2)	112 (56)	1	1

OR, odds ratio; AOR, adjusted odds ratio.

*P-value <0.05.

## Discussion

Job satisfaction among health extension workers plays a great role in implementing health extension programs (37). The study assesses the level of job satisfaction and its associated factors among health extension workers. This study revealed that the level of job satisfaction among study participants was 51.8%, with a 95% CI (46.97, 56.6%). Thus, approximately half of the study participants experienced dissatisfaction with their jobs. Factors such as earning more than 5,260 ETB as salary, living more than 10 km from the district town, receiving supportive supervision, and living with parents were significantly associated with job satisfaction.

According to this study, the level of HEW satisfaction in the study area was 51.8%. The finding from this study is consistent with the study conducted among HEWs in the Hadiya Zone, Southern Ethiopia (52.7%) (28) and the North Gonder Zone (56.2%) of the respondents (32). On the contrary, the result was greater than that of the studies conducted in the Harari region (44.2%) (20), West Ethiopia (41.46%) (28), Amhara Region (31.7%) (19), Sidama (36.6%) (29), eastern Ethiopia (38.5%) (38), Sri Lanka (23.7%) (39), Pakistan (41%) (22), Turkey (45.5%) (40),and the West Shoa Zone (34.9%) (20). This disparity is vividly attributed to the fact that there is local-based support and aid undertaken to boost their courage and initiatives, which utterly leads them to be satisfied with their job. However, our results fell short of the satisfaction rates reported in Tanzania (82.6%) and Malawi (71%) (41). This discrepancy might result from variations in a healthcare professional's socioeconomic characteristics, work environment, and organizational structure.

The findings from this study pointed out that distance from working health facilities was one of the predictors of job satisfaction. Thus, those health extension workers whose residential houses were located more than 10 km away from health facilities were 1.59 times more likely to be dissatisfied than their counterparts. This is what I found in Hadiya Zone (28). Explicitly, this is because the nearer the workplace, the easier and more suitable for the workers to be available at work within the intended time, enabling workers to avoid undesirable arguments with their bosses. More importantly, the workers can save their time and energy, so that they can get an opportunity to invest and spend their time on other activities instead of wasting time by traveling to their work. Hence, location disadvantages enormously affect job satisfaction.

Moreover, this study showed that the monthly salary of health extension workers is one of the independent predictors of job satisfaction. Thus, those HEWs who got a monthly salary >5,260 ETB were 1.69 times more likely to be satisfied than their counterparts. The result was in line with the study conducted in the North Wollo Zone (42) that indicated HEWs who have a low salary are more likely dissatisfied with their job and have the intention to leave their job. Having a better salary could satisfy and encourage the workers to remain in their jobs.

It is crystal clear that management issues play a pivotal and crucial role in determining the employee's level of satisfaction. Hence, to achieve and accomplish the intended organizational goals, there should be a sound and well-established leadership and management system. This study revealed that the level of satisfaction with management and supportive supervision in problem-solving was 62.89% and 52.28%, respectively. Moreover, those health extension workers who got supportive supervision were 1.64 times more likely to be satisfied than those who did not get supportive supervision from their supervisor. This is reinforced and supported by the study conducted in East Shoa Zone (37), Hadiya Zone (28), Addis Ababa (17), West Ethiopia (19), New Guinea (43), and Gondar. One of the possible justifications related to this might be that workers feel that there is someone who makes their presence felt in their work, which in turn develops in them a sense of ownership and fulfilment.

Finally, this study pointed out that health extension workers not living with their parents were significant predictors of job satisfaction. Accordingly, the odds of experiencing job satisfaction when the health extension workers were not living with their parents were 1.94 times more than the jobs of those living with their parents. This is in harmony with the study conducted in Gondar (32). Obviously, those health extension workers who did not live with their parents share joy and happiness at home, although they might be experiencing a very tough time at the workplace. This could reduce their stress and anxiety, which in turn contributes to their satisfaction.

### Strength and limitations of the study

The purpose of our study was to assess the level of job satisfaction and contributing factors among rural HEWs in the zone. The wider scope and higher sample size of the study might be its strengths. Despite this, this study had certain limitations that should be taken into account when interpreting the findings. However, it is important to consider the limitations of this study when interpreting the results. The qualitative components of determining the degree of work satisfaction among rural HEWs were also lacking in this study. Since this study only included rural HEWs, it may not be feasible to extrapolate the results to the whole healthcare workforce in the study area. Furthermore, it may be challenging to provide more meaningful information regarding work satisfaction and associated characteristics, as well as the effects of COVID-19 outbreaks, because the study only used a cross-sectional design.

### Practice implications

Policymakers and stakeholders can be informed about these emerging job satisfaction-related issues among HEWs' by using the study's findings to guide the development of programs aimed at establishing intervention tactics that may satisfy and motivate HEWs to expand health coverage and strengthen health extension programs in Ethiopia.

## Conclusion

Almost half of the health extension workers were dissatisfied with their jobs. Job satisfaction among HEWs was predicated on supportive supervision, remuneration, distance from closest health facility, and residence away from parents. To increase health coverage, strengthen health extension programs, and enhance service delivery, it is essential to develop intervention strategies that may satisfy and encourage HEWs.

## Data Availability

The original contributions presented in the study are included in the article/Supplementary Material, further inquiries can be directed to the corresponding author.
